# Mortality and Morbidity in a Population Exposed to Emission from a Municipal Waste Incinerator. A Retrospective Cohort Study

**DOI:** 10.3390/ijerph16162863

**Published:** 2019-08-10

**Authors:** Anna Maria Romanelli, Fabrizio Bianchi, Olivia Curzio, Fabrizio Minichilli

**Affiliations:** Unit of Environmental Epidemiology and Disease Registries, Institute of Clinical Physiology-IFC, National Research Council of Italy-CNR, Via G. Moruzzi, 1-56124 Pisa, Italy

**Keywords:** residential cohort study, waste, incinerator, mortality, hospitalization

## Abstract

In the present research, we evaluated the health effects of exposure to the municipal waste incinerator (MWI) in Pisa, Italy, through a population-based cohort design. The individual exposure pattern in the area was estimated through CALPUFF dispersion models of NO_χ_ (developed by Atmospheric Studies Group Earth Tech, Lowell, Massachusetts), used as pollution proxies of the MWI and the relevant industrial plant, and through land-use regression for NO_χ_ due to traffic pollution. Using Cox regression analysis, hazard ratios (HR) were estimated adjusting for exposure to other sources of pollution, age, and socioeconomic deprivation. An adjusted linear trend of HR (HRt) over the categories of exposure, with the relative 95% CI and p-value, was also calculated. Mortality and hospital discharge were studied as impact outcomes. Mortality analysis on males showed increased trends of mortality due to natural causes (HRt *p* < 0.05), the tumor of the lymphohematopoietic system (HRt *p* = 0.01), cardiovascular diseases (HRt *p* < 0.01); in females, increased trends for acute respiratory diseases (HRt *p* = 0.04). Morbidity analysis showed a HRt for lymphohematopoietic system tumor in males (HRt *p* = 0.04). Some of the excesses are in agreement with previous evidence on the health effects of MWIs, although the observation in males but not in females, suggests a cautious interpretation. Confounding due to other sources of exposure cannot be ruled out. The evidence was considered important in the decision-making process of the waste cycle.

## 1. Introduction

Incineration is a waste treatment process that involves the combustion of organic substances contained in waste materials. The thermal treatment of waste generates chemical substances that are considered as a potential health hazard to humans: metals (cadmium, thallium, zinc, mercury, chrome, arsenic, lead, cobalt, manganese, nickel, and vanadium), polycyclic aromatic hydrocarbons (PAH), fine and ultra-fine particulate, acids (fluoridric, chloridric), gases (SO_2_, NO_2_, CO), and polychlorinated products (polychlorinated biphenyls PCB, dioxins, furans). Inhalation (gases, particulate, PAHs), ingestion (PCB) and dermal contact (metals, PAH) are the recognized routes of exposure [1,2,3]. Waste incineration plants have been reported as a source of health damage to populations living in the vicinity of the plants, however, the scientific evidence is still limited due to the low number of studies carried out and as a result of various sources of uncertainty. Reviews of epidemiological studies have regarded incinerators operating in the past decades, mainly including studies with an ecological design, with exposure estimated through surrogate measures, such as distance from the plant, and often without or with inadequate control of potential confounders [2,4,5,6,7,8,9,10]. Studies on biomarkers show that populations exposed to emissions have higher biological levels of released substances compared with less exposed populations [7,8,9,10,11].

Although various uncertainties limit the overall interpretation of the findings, there is evidence that people living in proximity to an incinerator have an increased risk of all types of cancer [12,13], including stomach, colorectal, liver, renal, pleural and lung cancer, gallbladder and bladder for men, non-Hodgkin lymphoma and leukemia, and childhood-cancer/leukemia [13,14]. Studies on incinerators in France and in Italy have suggested an increased risk of non-Hodgkin lymphoma (NHL) [15], soft-tissue sarcoma [16,17], lung cancer [18], and neoplasia of the nervous system and liver [12]. Although the studies conducted by Shy et al. [19] and Lee and Shy [20] did not show respiratory effects. Other studies have reported increases in respiratory diseases or symptoms in populations residing near incinerators [21,22,23,24] and in children [25,26]. Other epidemiological studies on incinerators have shown an excess risk of cardiovascular diseases [21,23,24,27,28] and urinary diseases [21].

Some studies have highlighted excesses of tumors associated with exposure to dioxins [16,29] calling into question the high concentrations of these substances in the emissions released by old incinerators. In 2000, the European Community imposed limitations on permitted emissions (Directive 2000/76/EC, adopted in Italy in 2005) after which there was a gradual decrease in the concentrations emitted. In fact, the maximum permitted concentrations of dioxins, in Italy, have been lowered from 4000 ng/m^3^ (Ministerial Decree DM 7 December 1990) to 0.1 ng/m^3^ (Legislative Decree number 5 November 2005) [2]. Moreover, where a health impact of the change in technology has been reported, the results have been encouraging [30].

The proximity of other industrial plants and heavy traffic roads, and the presence of socio-economic disadvantaged groups within the exposed populations, are crucial issues in the health impact assessment of incinerators [31]. Advanced methods and tools to assess the weak associations between environmental exposures and health outcomes are thus valuable, especially when studying long-term latency diseases such as cancers and past exposure. Environmental research activity in this field requires conducting complex analysis because population health is influenced by interactions between individual and environmental characteristics, exposures and related health behaviors. 

The importance of using a flexible modeling strategy for assessing the risk of cancer in the vicinity of municipal solid waste incinerators was outlined by Goria et al. [32]. Dispersion models to assess the exposure to municipal solid waste incinerators reduce the exposure misclassification (with respect to distance), taking into account several factors, such as point source and meteorological characteristics, topography, and orography [33]. In recent epidemiological studies conducted in Italy, models for cohorts of geocoded residents were used, with individual exposure based on dispersion models and risk calculation adjusted for potential confounders, including environmental and occupational exposures and socio-economic deprivation [21,22]. The objective of the present epidemiological study is to assess the impact on the health of exposure to emissions produced by the municipal waste incinerator (MWI) in the municipality of Pisa, through a residential cohort study on mortality and hospital admissions. For this purpose, nosological causes associated with the main air pollutants were selected, and other sources of air pollution and indicators of socio-economic status were considered.

## 2. Methods

This study was set up in December 2014 as part of a convention between the municipality of Pisa and the Institute of Clinical Physiology of the National Research Council of Italy, IFC-CNR (Environmental Epidemiology and Disease Registries Unit). The research results were made public in October 2017. The methods were carried out in accordance with the relevant guidelines and regulations and in accordance with the Declaration of Helsinki Ethical Principles. No personal identifiers were sent to the research staff, all addresses were geocoded and the personal data were analyzed anonymously.

### 2.1. The Incinerator (MWI) and the Area of Investigation

**Figure 1:**
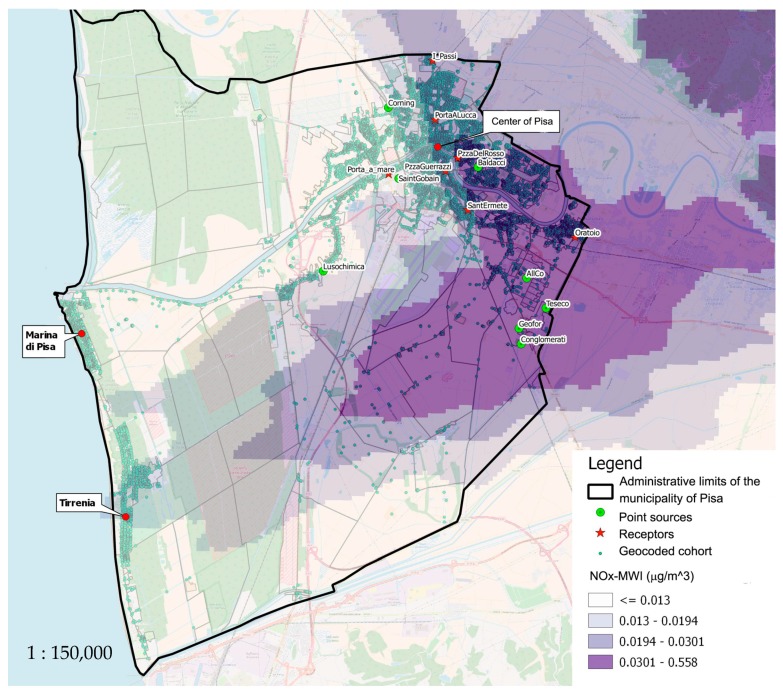
The study area.

The study area (Figure 1) is delimited by the administrative borders of the municipality of Pisa (89,158 inhabitants) in central Italy. The MWI is located in the peri-urban area outside of Pisa town center. The plant has been operative since 1981 and treats about 65,000 tonnes of waste per year. The MWI has been modified and renewed many times over the years in order to comply with the law. In 2000, the plant was equipped for energy recovery with a steam generator, turbines, and generator. Over time, different types of new filter bags have been used in order to improve the ability to eliminate dioxins. The use of catalytic filter bags facilitates the removal of micro-pollutants from 10 to 100 times below the limit and reduces the NO_χ_ concentration in the chimney. The latest generation of filter bags used are catalytic bags for NO_χ_, which, in particular, act on the structure of dioxins and furans that are degraded and destroyed.

Pisa has a predominance of tertiary and commercial activities, with three universities, a large research center, and a regional hospital. Industrial settlements have shrunk over the past few decades and thus only small factories remain, along with a medium-sized factory, Saint Gobain (Pisa, Italy), which produces glass. The area surrounding the MWI is located within a network of roads that lead to the city, which includes small industries, artisans’ workshops, an exhibition center, and agricultural activities. The study area (Figure 1) includes the greatest sources of pollution (such as the MWI, principal roads, and industrial plants) and for which environmental data produced by the regional agency for environmental protection (Pisa, Italy) are available.

### 2.2. Study Design

A residential cohort design with a retrospective approach was adopted. The cohort of residents is dynamic. The following were considered during the follow-up period (1 January 2001–31 December 2014): (a) migratory movements from and to the municipality, (b) migratory movements within the municipality, (c) births and deaths of residents in the municipality. For each subject in the cohort, the residential history during the follow-up period was considered, and each residence address was georeferenced. All subjects were followed up until the date of residence exit from the study area, the first specific-cause of hospitalization, or the time of death.

### 2.3. Exposure Assessment

Atmospheric dispersion models relating to emissions from the MWI and the other pollution sources located in the area were used by the CALMET-CALPUFF method (http://www.src.com/) carried out by the TEA (Energy and Environment Technologies) Engineering group, (http://www.tea-group.com/). The simulation models used environmental and meteorological data for 2015 and 2006, and considering the orography of the area. Annual average concentration maps for NO_χ_, PM_10_, SO_2,_ and selected metal (Hg, Zn, Cd) emissions were obtained. NO_χ_ was considered as a surrogate tracer of air pollution in order to estimate exposure to the MWI (called NO_χ_-MWI) and the other industrial plants (NO_χ_-IND). Despite the different chemical nature of the pollutants emitted in the area of investigation of the residential cohort, their diffusion in the air follows a common model. In fact, their diffusions were strongly correlated with each other (correlation coefficients ρ > 0.65 with type I error probability *p* < 0.001). Therefore, as a pollutant surrogate of the emissions in the present study the diffusion map of nitrogen oxides (NO_χ_) was used.

NO_χ_ was used as a surrogate of air pollution due to its high correlation with each of the other pollutants, and its availability for each source. For the other industrial plants, an overall NO_χ_ concentration map, defined as the sum of the NO_χ_ concentrations from each map, was used (called NO_χ_-IND). As no concentration maps were available for the major roads, a model of land-use regression for the estimation of traffic-related NO_χ_ (NO_χ_-TR) was used [34,35,36].

For each subject in the cohort, the history of residential exposure to NO_χ_ during the follow-up period was considered [23]. Each subject contributed to the study with an exposure load, determined by the sum of the time spent in each address in which the person had been resident (person-years). For each source of pollution, four exposure classes were defined using the quartiles of the distribution of NO_χ_ concentrations attributed to the cohort population. The four classes were so defined as follows: (1) low exposure NO_χ_-MWI ≤ 0.013; (2) medium-low exposure to pollution 0.013 < NO_χ_-MWI ≤ 0.019; (3) medium-high exposure to pollution 0.019 < NO_χ_-MWI ≤ 0.031; (4) high exposure NO_χ_-MWI > 0.03 (exposure classes of MWI in Figure 1).

### 2.4. Definition of Health Indicators

As health outcomes, mortality and hospital discharge records were considered. Data were used from the Tuscan Regional Mortality Register (Firenze, Italy ) for the period 2001–2012 and from the Regional Hospital Information System (Firenze, Italy ) (Hospital Discharge Records, HDRs) for 2001–2014. All record linkage procedures were carried out ensuring anonymity, according to the management rules of the regional information systems, in compliance with the current legislation on privacy. The causes of mortality and hospitalization were reported, which were defined a priori in line with the strongest evidence in the scientific literature in terms of air pollution, with the relative ICD IX codes in Table A1. In the Italian Health National Service, the HDRs are the information gathering tool for every patient discharged from public and private institutions throughout the national territory. The hospitalization data we used include hospitalization in ordinary (planned by appointment), day-hospital, and emergency regimens. For each hospitalization during the follow-up period, the only main diagnosis for the first discharge was considered.

### 2.5. Socio-Economic Status

The socio-economic status (SES) of each patient was expressed using the Italian deprivation index (DI) calculated using the 2001 Italian population census tract data [37]. DI is a sum of five census standardized continuous variables based on a regional average: low level of education, unemployment, one-parent family, home rental, and home overcrowding. TDI is an ordinal variable classified on the basis of the quintiles of the DI distribution in the cohort, with deprivation classes high, medium-high, medium, medium-low, and low.

### 2.6. Statistical Analysis

To study the relationship between the exposure of NO_χ_-MWI and the cause-specific mortality and morbidity of the residents in the area, a hazard ratio of MWI exposure adjusted for age, other environmental sources (NO_χ_-IND, NO_χ_-TR), and DI was calculated separately for men and women, by multiple Cox linear regression [hazard ratios with confidence interval at 95% of probability (HR, CI95%)]. HRs were calculated comparing each of the most exposed areas with the least exposed area, considered as a reference. The value of the probability of the first type error *p* < 0.05 was considered statistically significant (*p* < 0.10 was reported as borderline significant). Meteorological factors such as season, temperature, and humidity were not included in the Cox regression model since they were duly considered in the model for the environmental study that estimated the diffusion of pollutants precisely taking into account the orography and meteorology characteristics. 

For each cause of death and hospitalization, an adjusted linear trend of HR (HRt) over the categories of exposure with the relative 95% CI and *p*-value was also calculated. The analyses were performed using STATA release 13 (Stata Statistical Software, College Station, TX: Stata Corporation, USA). 

## 3. Results

The characteristics of the study population are reported in Table 1. The cohort included 132,293 inhabitants (1,092,817 person-years, of which 52.6% were women). A total of 335,760 person-years (30.72%) were included in the class with the highest exposure.

The mean Municipal Waste Incinerator related NO_χ_ (NO_χ_-MWI) distribution as estimated by the diffusion model was 0.025 µg/m^3^ (SD 0.019 µg/m^3^), with a maximum value of 0.522 µg/m^3^. The mean concentration level in the four classes was: lowest class: NO_χ_-MWI (µg/m^3^) ≤ 0.013; second class: 0.013 < NO_χ_-MWI (µg/m^3^) ≤ 0.019; third class: 0.019 < NO_χ_-MWI µg/m^3^) ≤ 0.031; highest class: NO_χ_-MWI (µg/m^3^) > 0.031.

The association between sex and NO_χ_-MWI was statistically significant: in females, the highest percentages of person-years (PY) were in the second and third classes of NO_χ_-MWI, whereas in males, this percentage was higher in the third and fourth classes. The percentage of person-years (PY) increases across the classes of NO_χ_-MWI in, both, males and females. The association between age and NO_χ_-MWI was statistically significant: the average age decreased with the increase in the exposure class of NO_χ_-MWI. The association between other industrial plants related NO_χ_ (NO_χ_-IND) and NO_χ_-MWI was statistically significant: the averages of NO_χ_-IND were higher in the second and third classes than the other classes. The association between traffic-related NO_χ_ (NO_χ_-TR) and NO_χ_-MWI was statistically significant: the NO_χ_-TR averages were higher in the third and fourth classes than the other classes (the LUR model, estimated as part of the project ESCAPE, 2013, estimates NO_χ_ emissions from road traffic that are higher than those of the incinerator, but differently distributed over the territory). The DI class averages were significantly heterogeneous among the NO_χ_ classes. The mortality analysis showed (Table 2): in males, an excess general mortality in the highest exposure class compared to the lowest class (n. 1,662; HR = 1.09; 95% CI 1.00–1.19) and an HR trend (HRt = 1.02; *p* = 0.06), and in particular mortality due to natural cause (n. 1,571; HR = 1.10; 95% CI 1.01–1.20; HRt = 1.03; *p* = 0.05); an excess risk and an HR trend in males with lymphohematopoietic system cancers (n. 59; HR = 1.79; 95% CI 1.03–3.12; HRt = 1.23; *p* = 0.01); an excess risk and a HR trend in males for cardiovascular diseases (n. 592; HR = 1.21; 95% CI 1.05–1.39; HRt = 1.06; *p* = 0.01); in particular for acute myocardial infarction and ischemic diseases of the heart (n. 104; HR = 1.38; 95% CI 0.97–1.98; HRt = 1.17; *p* = 0.01; n. 244; HR = 1.24; 95% CI 0.99–1.55; HRt = 1.09; *p* = 0.01 respectively); an excess risk and an HR trend in females of the highest class of exposure for acute respiratory diseases (n. 39; HR = 2.52; 95% CI 1.31–4.83; HRt = 1.19; *p* = 0.04); an excess risk and an HR trend in males for tumors of the lymphohematopoietic system (n. 59, HR = 1.79 95% CI 1.03–3.12; HRt = 1.23, *p* = 0.01); in particular, an HR trend in both genders for leukemia although these were based on few cases (Male HRt = 1.26; *p* = 0.06–Female, HRt = 1.39; *p* = 0.06) and an HR trend in males for non-Hodgkin Lymphoma (HRt = 1.29; *p* = 0.08). The morbidity analysis (Table 3) showed an HR trend for tumors of the lymphohematopoietic system in males (HRt = 1.14; *p* = 0.04). In addition, a trend close to the limit of statistical significance was observed for non-Hodgkin lymphoma in males (HRt = 1.21; *p* = 0.06) and leukemia in males (HRt = 1.21; *p* = 0.07).

## 4. Discussion

This study assessed the health effects of the exposure to pollutants emitted by the MWI. The mortality and morbidity were studied in a cohort of residents in the municipality of Pisa in the period 2001–2014. The study was conducted with the retrospective longitudinal residential cohort approach in which residents were followed over fourteen years by assigning a level of exposure to the address of residence of each subject which was obtained by modeling techniques based on dispersion models. The residential cohort approach is the highest level of etiological study design for which it is possible to use routine health data. The objective was to assess whether and to what extent the risks of death/hospitalization of residents were greater where the exposure to NO_χ_-MWI was higher.

Exposure to NO_χ_ produced by MWI was chosen as a surrogate measure of exposure to air pollution, which was supported by the high and statistically significant correlation between NO_χ_, PM, SO_2_, and selected metals. The NO_χ_ simulation models used environmental data from 2015 and meteorological data from 2006. The assumption of a lack of temporal variability of exposure is sustainable considering that major changes in operating conditions of the MWI plant in 2001–2014 are not documented by its monitoring systems, and as the meteorological data for 2006 was in the middle of the follow-up period, it can be reasonably assumed as representative. The quartiles of the spatial distribution of the NO_χ_-MWI were used to define four classes of exposure without considering the absolute values of the annual NO average, in order to divide the total area into four subareas according to an ordinal exposure scale.

A limitation of the present epidemiological study is to have estimated the environmental exposure based on the concentration attributed to the residence address, through a relapse model (as a proxy). Moreover, the study design did not consider individual risk factors, such as occupational activity, workplace, tobacco smoke, and other lifestyle habits, which are recognized as potential confounders as they are associated with both the exposure and health outcomes under study. In fact, data on the personal habits of subjects that might have played a role in the investigated health outcomes (in particular cigarette smoking, alcohol use, obesity, physical activity, and occupation) were not available, and considering the retrospective nature of the study, collecting such information would have been practically impossible. Our assumption that these risk factors are uniformly distributed in the study area seems reasonable considering that the complex exposure map was defined by the diffusion model. On the other hand, an alternative hypothesis that the confounders are greater in the most exposed areas would seem implausible.

Although a distortion effect due to lack of adjustment for the above mentioned potential confounders cannot be ruled out, assuming a positive role of the correction applied by the deprivation index is reasonable. In fact, many of the personal habits listed are associated with socioeconomic status and DI was considered as a proxy of individual socio-economic deprivation. Research on the relation between socioeconomic status, neighborhood environments, and health has grown rapidly in recent years. Neighborhood deprivation, often measured as a composite of sociodemographic census-level variables, is one way of measuring the socioeconomic environment. For example, living in a more socioeconomically deprived neighborhood has been associated with higher smoking levels [38,39,40]. The relationship between socioeconomic status and health is due to behavioral factors, such as unhealthy eating habits [41] or specific unhealthy drinking patterns [42].

Our approach was in line with other studies of national and international importance [43,44,45,46,47,48,49,50,51] and does not rule out that there may still be residual confounding factors. On the other hand, the assumption that the effect of non-controllable residual confounding is non-differential and consequently leads to a risk underestimation seems reasonable. The selection of just a limited number of causes of mortality and hospitalization with stronger evidence of association with environmental determinants weakens the possible distortion effect of multiple testing.

The present study shows associations between exposure to MWI emissions and an increased mortality trend among males for general and natural causes (trend + 2% and + 3%, respectively), tumor of the lymphohematopoietic system (trend + 23%), in particular NHL (+ 29%), cardiovascular diseases (+ 6%); acute respiratory diseases among females (+ 19%); and leukemia in both genders (M + 26%, F + 39%). In addition, an increasing HR trend was confirmed for hospitalization due to tumor of the lymphohematopoietic system in males (+ 14%), in particular for NHL (+ 21%) and leukemia (+ 21%).

In general, the excesses detected for cardiovascular and respiratory diseases are in agreement with the epidemiological evidence available on the cardiovascular and respiratory effects induced by air pollution [52,53,54,55,56,57,58,59,60]. Excess risks for cardiovascular diseases have been reported in relation to atmospheric pollution due to MWIs [21,23,24,27,28] and respiratory diseases [9,21,22,23,24]. Exposure to incinerators has been variously associated with increases in both mortality and hospitalization for tumors of the lymphohematopoietic system, in particular, NHL and leukemia [7,8,13,15,61,62].

The MWI of Pisa has been operative since 1981 and has subsequently undergone many changes and renewals adapting to the emissions law regulations. This variability in the emissions over time has advised us against a time trend study in favor of a comparison study between exposed and non-exposed over a long period. The follow-up of fewer than 20 years of this study did not fully cover the induction-latency period of most cancers. The greater number of risk associations for mortality compared to hospitalization could be partly due to exposures occurring at the beginning or before the start of the follow-up, and a more extensive examination regarding the age of the end-points would be useful.

The excess mortality and hospital admissions for tumors of the lymphohematopoietic system observed only among men in areas with higher pollution estimates, as well as the mortality excess for diseases of the cardiovascular system, require further investigation. In the light of the misalignment observed between males and females, the present results should be evaluated with caution, also because women are generally a more stable population than men and therefore less exposed to misclassification of exposure [30]. Gender differences in health risk features should be explored further also examining behavioral risk factors of men and women and the consequences in terms of health inequalities [63].

## 5. Conclusions

The results achieved were sent to the administrators of the municipality of Pisa with the general recommendation that the health of all the population residing in areas adjacent to the incinerator should be protected. The present epidemiological study recommends the adoption of specific policies to contain both the production of waste and the need to dispose of it. We suggested caution in deciding whether to build a new incinerator or to renovate the existing one, taking into consideration the current state of health in the relapse area. Population health is affected by complex interactions between different determinants as socio-economic, environmental, and policy circumstances that can directly influence health outcomes, affect exposure (confounding), or the relationships between environment and health (effect modifying). Although our study used a cohort design based on the individual exposure data (proxy) and health effect with adjustment by socio-economic status indicator, we are aware that other variables would be relevant to achieve a more reliable assessment. We are aware that improving the definition of exposure and potential confounders is the mainstream to increase the reliability of the evidence and their transferability to public health policies. We are advocates of the holistic approach but often we find ourselves having to mediate between our aspirations and the limits of data and available resources, in addition to having to guarantee the respect for the privacy of the people involved in the studies. The adoption of a specifically designed environmental and health surveillance system and the true involvement of stakeholders in all phases of waste management have also been suggested. At the end of 2017, the results of the study were presented to the municipal administrators and then in a public assembly. Since the beginning of 2018, the incinerator has been shut down following the decision to decommission it and decisions are now being taken regarding the entire waste cycle.

## Figures and Tables

**Table 1:** Characteristics of the cohort by NO_χ_-MWI classes.

	Classes of NO_χ_-MWI (µg/m^3^)				Total	*p*
Cohort	NO_χ_-MWI ≤ 0.013	0.013 < NO_χ_-MWI ≤ 0.019	0.019 < NO_χ_-MWI ≤ 0.031	NO_χ_-MWI > 0.031		

Female (PY)	102,130	135,534	161,875	175,633	575,172	<0.001
Females in the exposure classes (%)	17.76	23.56	28.14	30.54	100	
Females in single exposure class (%)	52.18	53.10	52.89	52.31	52.63	
Male (PY)	93,613	119,694	144,211	160,127	517,645	
Males in the exposure classes (%)	18.08	23.12	27.86	30.93	100	
Males in single exposure class (%)	47.82	46.90	47.11	47.69	47.37	
Total (PY)	195,743	255,228	306,086	335,760	1,092,817	
Subjects in the exposure classes (%)	17.91	23.36	28.01	30.72	100	
Subjects in single exposure class (%)	100	100	100	100	100	
Age (mean)	47.74	46.48	46.97	45.58	46.57	<0.001
SD	23.01	23.06	23.03	22.27	22.82	<0.001
NO_χ_-IND (mean)	0.86	1.50	1.27	0.76	1.10	<0.001
SD	0.62	0.95	0.35	0.31	0.66	<0.001
NO_χ_-LUR (mean)	26.56	31.01	35.14	32.14	31.72	<0.001
SD	10.35	10.79	12.13	12.32	11.94	<0.001
DI class (mean)	2.51	2.42	2.29	2.76	2.50	<0.001
SD	1.14	1.11	1.06	1.09	1.11	<0.001

Legend: MWI: Municipal waste incinerator; PY person-year; LUR: land use regression; DI: deprivation index.

**Table 2:** 2001–2012 mortality associated with exposure to NO_χ_-MWI for sex.

Mortality Causes	Sex	Exp Class *	N	Hazard Ratio **	*p*	IC_95%_		HR Trend	*p*	IC_95%_	
All causes	M	low	1114								
		high	1662	1.09	0.04	1.00	1.19	1.02	0.06	1.00	1.05
	F	low	1252								
		high	1728	1.03	0.42	0.95	1.12	1.01	0.26	0.99	1.04
Natural causes	M	low	1050								
		high	1571	1.10	0.03	1.01	1.20	1.03	0.05	1.00	1.05
	F	low	1213								
		high	1651	1.02	0.59	0.94	1.11	1.01	0.38	0.99	1.04
Stomach cancer	M	low	18								
		high	28	0.80	0.52	0.41	1.57	0.95	0.63	0.77	1.17
	F	low	9								
		high	18	1.18	0.72	0.49	2.80	1.08	0.51	0.85	1.38
Colorectal cancer	M	low	33								
		high	54	0.92	0.74	0.57	1.48	1.00	0.97	0.87	1.15
	F	low	42								
		high	62	1.02	0.92	0.66	1.59	1.00	0.95	0.88	1.15
Liver cancer	M	low	24								
		high	37	1.15	0.62	0.65	2.05	0.99	0.90	0.84	1.17
	F	low	24								
		high	29	0.90	0.74	0.48	1.67	0.91	0.37	0.74	1.12
Lung cancer	M	low	130								
		high	172	0.92	0.53	0.71	1.19	0.96	0.35	0.89	1.04
	F	low	36								
		high	57	1.22	0.39	0.77	1.94	1.05	0.51	0.91	1.21
Connective and other soft tissues	M	low	2								
		high	2	0.97	0.98	0.11	8.36	0.95	0.87	0.50	1.79
	F	low	2								
		high	1	0.27	0.36	0.02	4.58	0.85	0.68	0.39	1.86
Lymphatic and hematopoietic tissues	M	low	21								
		high	59	1.79	0.04	1.03	3.12	1.23	0.01	1.05	1.43
	F	low	23								
		high	38	1.26	0.43	0.71	2.21	1.09	0.34	0.92	1.29
Leukemia	M	low	10								
		high	26	1.64	0.23	0.73	3.70	1.26	0.06	0.99	1.61
	F	low	3								
		high	14	3.29	0.08	0.87	12.50	1.39	0.06	0.99	1.93
Non-Hodgkin lymphoma	M	low	5								
		high	20	2.31	0.12	0.80	6.68	1.29	0.08	0.97	1.70
	F	low	9								
		high	13	1.37	0.50	0.55	3.41	1.07	0.61	0.82	1.41
Central nervous system	M	low	4								
		high	13	1.87	0.32	0.54	6.44	1.22	0.20	0.90	1.65
	F	low	13								
		high	8	0.38	0.06	0.14	1.05	0.81	0.16	0.60	1.09
Cardiovascular diseases	M	low	372								
		high	592	1.21	0.01	1.05	1.39	1.06	0.01	1.02	1.11
	F	low	538								
		high	740	1.02	0.82	0.90	1.15	1.02	0.23	0.99	1.06
Acute myocardial infarction	M	low	58								
		high	104	1.38	0.07	0.97	1.98	1.17	0.01	1.05	1.31
	F	low	56								
		high	73	1.13	0.54	0.76	1.69	1.02	0.75	0.91	1.15
Ischemic heart diseases	M	low	153								
		high	244	1.24	0.06	0.99	1.55	1.09	0.01	1.02	1.17
	F	low	163								
		high	225	1.04	0.75	0.82	1.31	1.04	0.26	0.97	1.11
Cerebrovascular diseases	M	low	110								
		high	158	1.10	0.50	0.84	1.43	1.04	0.40	0.95	1.12
	F	low	172								
		high	248	1.11	0.35	0.89	1.38	1.03	0.34	0.97	1.10
Respiratory diseases	M	low	102								
		high	130	0.95	0.74	0.71	1.27	0.99	0.82	0.91	1.08
	F	low	86								
		high	111	1.14	0.43	0.82	1.58	0.99	0.90	0.90	1.09
Acute respiratory diseases	M	low	25								
		high	18	0.53	0.07	0.26	1.04	0.81	0.05	0.66	1.00
	F	low	18								
		high	39	2.52	0.01	1.31	4.83	1.19	0.04	1.00	1.41
Chronic pulmonary diseases	M	low	49								
		high	73	1.18	0.42	0.79	1.76	1.05	0.42	0.93	1.18
	F	low	47								
		high	48	0.87	0.56	0.55	1.39	0.94	0.36	0.82	1.08

* Exposure classes of MWI: low exposure NO_χ_-MWI ≤ 0.013; high exposure NO_χ_-MWI > 0.03; ** hazard ratio of MWI exposure adjusted for age, other environmental sources (NO_χ_-IND, NO_χ_-TR), and deprivation index was calculated separately for men and women by multiple Cox linear regressions comparing each of the most exposed areas with the least exposed area, considered as a reference.

**Table 3:** 2001–2014 hospitalization associated with exposure to NO_χ_-MWI for sex.

Causes	Sex	Exp Class *	N	HR **	*p*	IC_95%_		HR Trend	*p*	IC_95%_	
Cardiovascular diseases	M	low	1367								
		high	2080	1.00	0.96	0.93	1.08	1.00	0.70	0.98	1.03
	F	low	1187								
		high	1744	1.01	0.88	0.93	1.09	1.01	0.51	0.98	1.03
Ischemic heart diseases	M	low	484								
		high	728	0.98	0.77	0.86	1.12	1.01	0.67	0.97	1.05
	F	low	258								
		high	383	1.01	0.90	0.85	1.21	1.02	0.45	0.97	1.08
Acute myocardial infarction	M	low	231								
		high	373	1.08	0.42	0.90	1.30	1.03	0.27	0.98	1.09
	F	low	138								
		high	193	0.96	0.75	0.75	1.23	1.01	0.88	0.93	1.08
Cerebrovascular diseases	M	low	414								
		high	607	0.98	0.74	0.85	1.12	1.00	0.91	0.96	1.04
	F	low	413								
		high	611	1.00	0.95	0.87	1.15	1.01	0.54	0.97	1.06
Respiratory diseases	M	low	779								
		high	1200	0.95	0.35	0.86	1.05	0.99	0.35	0.96	1.02
	F	low	652								
		high	984	0.94	0.25	0.84	1.05	0.98	0.14	0.94	1.01
Chronic pulmonary diseases	M	low	97								
		high	148	1.12	0.42	0.84	1.50	1.00	0.99	0.92	1.09
	F	low	88								
		high	115	0.86	0.35	0.63	1.18	0.97	0.55	0.89	1.07
Acute respiratory diseases	M	low	276								
		high	408	0.91	0.28	0.77	1.08	0.95	0.07	0.91	1.00
	F	low	207								
		high	355	1.08	0.45	0.89	1.30	1.00	0.97	0.95	1.06
Stomach cancer	M	low	37								
		high	46	0.77	0.30	0.48	1.26	0.92	0.26	0.79	1.07
	F	low	15								
		high	31	1.30	0.45	0.66	2.56	1.06	0.56	0.87	1.29
Colorectal cancer	M	low	122								
		high	189	0.95	0.71	0.74	1.22	1.00	0.96	0.92	1.08
	F	low	131								
		high	163	0.83	0.16	0.64	1.08	0.99	0.80	0.92	1.07
Connective and other soft tissues	M	low	7								
		high	7	0.76	0.64	0.23	2.46	0.90	0.54	0.64	1.27
	F	low	6								
		high	5	0.72	0.63	0.19	2.71	0.72	0.11	0.48	1.08
Liver cancer	M	low	38								
		high	57	1.11	0.67	0.69	1.76	0.95	0.50	0.83	1.10
	F	low	32								
		high	40	0.94	0.82	0.55	1.60	0.94	0.49	0.79	1.12
Lung cancer	M	low	136								
		high	173	0.87	0.27	0.67	1.12	0.96	0.32	0.89	1.04
	F	low	40								
		high	78	1.34	0.17	0.88	2.05	1.11	0.11	0.98	1.26
Lymphatic and hematopoietic tissue	M	low	39								
		high	86	1.41	0.12	0.92	2.15	1.14	0.04	1.01	1.29
	F	low	38								
		high	71	1.21	0.39	0.78	1.88	1.05	0.42	0.93	1.19
Non-Hodgkin lymphoma	M	low	11								
		high	35	1.85	0.11	0.88	3.89	1.21	0.06	0.99	1.49
	F	low	14								
		high	28	1.54	0.24	0.76	3.12	1.12	0.25	0.92	1.37
Leukemia	M	low	13								
		high	34	1.75	0.13	0.86	3.57	1.21	0.07	0.98	1.49
	F	low	10								
		high	27	1.35	0.46	0.61	3.00	1.04	0.70	0.84	1.30
Central nervous system	M	low	26								
		high	44	0.99	0.98	0.58	1.72	1.02	0.85	0.86	1.19
	F	low	39								
		high	46	0.81	0.38	0.51	1.30	0.91	0.19	0.79	1.05

* Exposure classes of MWI: low exposure NO_χ_-MWI ≤ 0.013; high exposure NO_χ_-MWI > 0.03; ** hazard ratio of MWI exposure adjusted for age, other environmental sources (NO_χ_-IND, NO_χ_-TR), and Deprivation index was calculated separately for men and women by multiple Cox linear regressions comparing each of the most exposed areas with the least exposed area, considered as a reference.

## Appendix A

**Table A1:** Mortality and hospitalization causes considered in the present study-International Classification of Diseases, 9th Revision (ICD-IX) associated codes.

Mortality and Hospitalization Causes	ICD-9 Codes
All mortality causes	001–999
Mortality due to natural causes	001–799
Cancers	
Stomach	151
Colorectal	153–154
Liver cancer	155
Lung cancer	162
Connective and other soft tissues	171
Central nervous system	191–192, 225
Lymphatic and haematopoietic tissues	200–208
Leukemia	204–208
Non-Hodgkin Lymphoma	200, 202
Cardiovascular diseases	390–459
Acute myocardial infarction	410
Ischemic heart diseases	410–414
Cerebrovascular diseases	430–438
Respiratory diseases	460–519
Acute respiratory diseases	460–466, 480–487
Chronic pulmonary diseases	491–492, 494–496
